# Cost-effectiveness analysis of pyrotinib combined with trastuzumab and docetaxel as first-line treatment for HER2-positive metastatic breast cancer in China

**DOI:** 10.3389/fphar.2026.1845724

**Published:** 2026-06-25

**Authors:** Miao Liang, Sisi Liu, Xiaoxu Zhang, Qianna Liu

**Affiliations:** Department of Pharmacy, Hebei Medical University Third Hospital, Shijiazhuang, Hebei, China

**Keywords:** cost-effectiveness, first-line treatment, metastatic breast cancer, pyrotinib, tyrosine kinase inhibitor

## Abstract

**Objective:**

This study assessed the cost-effectiveness of pyrotinib combined with trastuzumab and docetaxel as a first-line treatment for HER2-positive metastatic breast cancer from the perspective of the Chinese healthcare system.

**Methods:**

A partitioned survival model was constructed to evaluate the cost-effectiveness of this regimen in patients with HER2-positive metastatic breast cancer. The primary outcomes were total costs, quality-adjusted life-years (QALYs), and the incremental cost-effectiveness ratio (ICER). The treatment strategy was considered cost-effective when the ICER fell below a predefined willingness-to-pay (WTP) threshold. One-way and probabilistic sensitivity analyses were conducted to address parameter uncertainties. Additional scenario analyses were performed to explore how different assumptions influenced the ICER.

**Results:**

In the base-case analysis, the pyrotinib group incurred total costs of $136,400.69 compared with $83,887.55 in the placebo group. The corresponding effectiveness was 5.20 QALYs *versus* 3.89 QALYs. The ICER was $40,245.81/QALY, which exceeded the WTP threshold (two times China’s per capita GDP in 2025, $27,906.03/QALY). One-way sensitivity analysis revealed that utility of progression-free survival and the price of pyrotinib were the most influential factors on the ICER. Probabilistic sensitivity analysis indicated that at the WTP threshold of $27,906.03/QALY, pyrotinib had a 6.1% probability of being cost-effective. Scenario analyses confirmed the robustness of the model. Price threshold analysis showed that the price of pyrotinib would need to be reduced by more than 34.45% to bring the ICER below the WTP threshold.

**Conclusion:**

From the Chinese healthcare perspective, pyrotinib combined with trastuzumab and docetaxel may not be cost-effective for HER2-positive metastatic breast cancer under current prices and the WTP threshold. A price reduction would be required to achieve cost-effectiveness.

## 1 Introduction

Breast cancer is the most common malignant tumor in women and poses substantial challenges to global health, society, and the economy. In 2022, approximately 2.3 million new breast cancer cases and 666,103 related deaths were reported worldwide, accounting for 11.5% of all new malignant tumor cases (Kim et al., 2025). Some projections estimate that the global number of breast cancer cases will exceed 6 million by 2050 (Freihat et al., 2025). Human epidermal growth factor receptor 2 (HER2) overexpression or gene amplification occurs in approximately 20% of breast cancer cases (Kunte et al., 2020). HER2 positive status is associated with enhanced tumor aggressiveness and an unfavorable prognosis. The introduction of targeted therapy has revolutionized the treatment of HER2 positive breast cancer (Oh and Bang, 2020). Trastuzumab specifically binds to the HER2 receptor and blocks its downstream signaling pathways. In addition, it enhances the killing effect of immune cells on tumor cells through antibody dependent cell mediated cytotoxicity (ADCC) (Hurvitz et al., 2023), thereby establishing its role as a cornerstone drug for HER2 positive breast cancer. However, clinical practice has shown that long term use of single targeted agents tends to induce drug resistance mechanisms, such as receptor mutations and bypass activation of signaling pathways, leading to disease progression in some patients within one to 2 years of treatment (Li et al., 2025). Therefore, exploring combined targeted therapeutic strategies that involve multiple targets and diverse mechanisms has become a key direction to overcome the bottleneck of drug resistance.

Pyrotinib is an orally administered irreversible pan-HER tyrosine kinase inhibitor (TKI) that targets EGFR, HER2, and HER4. It blocks the downstream PI3K/Akt and Ras/MAPK signaling pathways and inhibits tumor proliferation, invasion, and metastasis (Kunte et al., 2020; Yuan et al., 2025). Interim findings from the phase 3 PHILA trial demonstrated that pyrotinib combined with trastuzumab and docetaxel significantly improved progression free survival (PFS) compared with the control group (24.3 months versus 10.4 months; hazard ratio [HR] 0.41), with a manageable safety profile. This regimen provided the first evidence that a dual anti-HER2 strategy combining a monoclonal antibody and a small molecule tyrosine kinase inhibitor could serve as first-line therapy for HER2 positive metastatic breast cancer (Ma et al., 2023). Based on these results, the National Medical Products Administration of China approved this regimen for first-line treatment of HER2 positive metastatic breast cancer in April 2023. In March 2026, the PHILA study updated its long-term survival data (Ma et al., 2026). The study confirmed that pyrotinib plus trastuzumab and docetaxel significantly prolonged PFS in patients with HER2 positive metastatic breast cancer compared with the placebo containing combination (22.1 months vs 10.5 months; HR 0.44; one sided nominal P < 0.001) and reduced the risk of death by 36% (HR 0.64). At 5 years of follow up, 66% of patients were alive and 30% remained free of disease progression or death. No new safety concerns were observed.

Cancer related disease burden remains an important public health problem in China (Wu et al., 2025). Breast cancer is one of the most common malignancies in Chinese women. The patient population is large, and total treatment expenditure continues to increase. For patients with HER2 positive metastatic breast cancer, long term treatment is often required after the disease reaches an advanced stage. Targeted drugs and combination regimens are costly. Post progression management, adverse event (AE) treatment, and follow up monitoring further increase medical expenditure (Wu et al., 2024; Zhou et al., 2024). One study showed that economic factors were the main reason why Chinese patients with HER2 positive breast cancer refused targeted therapy (Wang et al., 2023). Given limited healthcare resources and medical insurance funds in China, improving the efficiency of resource allocation has become a key issue in cancer treatment decision-making. The National Reimbursement Drug List Negotiation (NRDLN) is an important measure to improve the accessibility and affordability of innovative drugs (Zhu H. et al., 2022; Li B. X. et al., 2024). Pharmacoeconomic evaluation has become an important type of evidence for medical insurance access and price negotiation in China. Cost-effectiveness analysis can provide direct economic evidence (Liu et al., 2022; Zhang et al., 2022). Therefore, conducting cost-effectiveness studies of new combination regimens for HER2 positive metastatic breast cancer is of practical significance for supporting medical insurance access policies in China, optimizing healthcare resource allocation, and improving patient access to treatment.

To date, no cost-effectiveness analysis has been conducted on pyrotinib plus trastuzumab and docetaxel as first-line therapy for HER2-positive metastatic breast cancer. From the Chinese healthcare system perspective, we assessed the cost-effectiveness of this regimen using long-term survival data from the PHILA trial, aiming to provide evidence for clinical practice and healthcare policy development in China.

## 2 Methods

### 2.1 Target patient cohort and interventions

The study population and treatment regimen were derived from the phase III PHILA study, a multicenter, double blind, randomized controlled phase III clinical trial conducted across 40 centers in China. Eligible patients were aged 18–75 years with pathologically confirmed HER2 positive metastatic breast cancer and had received no prior systemic treatment for metastatic disease.

Patients were randomized in a 1:1 ratio to receive either oral pyrotinib 400 mg once daily or placebo, in combination with intravenous trastuzumab and docetaxel. Trastuzumab was administered at 8 mg/kg in the initial cycle and 6 mg/kg in subsequent cycles. All treatments were given on day one of each 21-day cycle. The pyrotinib dosage could be sequentially reduced from 400 mg to 320 mg and then to 240 mg. The docetaxel dosage could be reduced to a minimum of 60 mg/m^2^. Patients were required to receive at least six cycles of docetaxel, with the median treatment duration set at eight cycles. Treatment continued until disease progression, unacceptable toxicity, withdrawal of informed consent, investigator decision to terminate, or patient death. Upon disease progression, patients received subsequent systemic antitumor therapies according to the Chinese Society of Clinical Oncology Guidelines for the Diagnosis and Treatment of Breast Cancer (2026 Edition) (The Society of BreastCancer China Anti-Cancer Association, 2026). These subsequent therapies included anti-HER2 agents, HER2 tyrosine kinase inhibitors, HER2 antibody drug conjugates, and chemotherapy. Common chemotherapy regimens for HER2 positive breast cancer include anthracyclines combined with cyclophosphamide followed by taxane sequencing. The proportion of patients receiving different therapies was based on data reported in the PHILA trial (Supplementary Tables S1, S2). Patients who did not receive second line therapy were assumed to be managed with best supportive care (BSC) (Table 1).

**TABLE 1:** Key model parameters.

Parameter	Estimate	Range		Distribution	Reference
		Minimum	Maximum		
Survival curve parameters					
OS of pyrotinib: Log-normal	meanlog = 4.561,sdlog = 1.165			Model fitting	
OS of placebo: Log-logistic	shape = 1.465,scale = 72.061			Model fitting	
PFS of pyrotinib: Log-logistic	shape = 1.358,scale = 25.827			Model fitting	
PFS of placebo: Log-logistic	shape = 1.975,scale = 11.420			Model fitting	
Cost of drug ($)					
Pyrotinib (80 mg)	9.14	7.31	10.97	Gamma	yaozh.com
Trastuzumab (150 mg)	220.50	176.40	264.60	Gamma	yaozh.com
Docetaxel (20 mg)	6.7	5.36	8.04	Gamma	yaozh.com
Cost of subsequent treatment ($)					
Pertuzumab (420 mg)	554.54	443.63	665.44	Gamma	yaozh.com
Inetetamab (50 mg)	82.6	66.08	99.12	Gamma	yaozh.com
Trastuzumab emtansine (100 mg)	501.20	400.96	601.44	Gamma	yaozh.com
Trastuzumab deruxtecan (100 mg)	967.67	774.14	1161.21	Gamma	yaozh.com
Cyclophosphamide (200 mg)	3.36	2.69	4.03	Gamma	yaozh.com
Doxorubicin (10 mg)	2.39	1.91	2.87	Gamma	yaozh.com
Paclitaxel Injection (30 mg)	5.82	4.66	6.98	Gamma	yaozh.com
Costs of follow-up care per cycle ($)	43.21	34.57	51.85	Gamma	local price
Cost of BSC per cycle ($)	790.03	632.02	948.04	Gamma	Zhu et al. (2022b)
Cost of terminal care per patient ($)	1,901.59	1521.27	2281.91	Gamma	Zhu et al. (2026)
Cost of managing AE (grade>3) per event($)					
Neutropenia	387.19	309.75	464.62	Gamma	Jin and Li (2025)
Leukopenia	543.69	434.95	652.42	Gamma	Jin and Li (2025)
Diarrhea	118.30	94.64	141.96	Gamma	local price
Hypokalemia	32.20	25.76	38.64	Gamma	local price
Anemia	510.30	408.24	612.36	Gamma	Zhu et al. (2026)
Vomiting	46.73	37.38	56.08	Gamma	Chen et al. (2021)
Receiving at least one anti-tumor therapies in pyrotinib group (%)	58.6	46.88	70.32	Beta	Ma et al. (2026)
Receiving best supportive care in pyrotinib group (%)	41.4	33.12	49.68	Beta	Ma et al. (2026)
Receiving at least one anti-tumor therapies in placebo group (%)	78.2	62.56	93.84	Beta	Ma et al. (2026)
Receiving best supportive care in placebo group (%)	21.8	17.44	26.16	Beta	Ma et al. (2026)
Probabilities of AEs in pyrotinib group (%)					
Neutropenia	63	50.4	75.6	Beta	Ma et al. (2026)
Leukopenia	53.2	42.56	63.84	Beta	Ma et al. (2026)
Diarrhea	47.8	38.24	57.36	Beta	Ma et al. (2026)
Hypokalemia	15.8	12.64	18.96	Beta	Ma et al. (2026)
Anemia	11	8.8	13.2	Beta	Ma et al. (2026)
Vomiting	7.4	5.92	8.88	Beta	Ma et al. (2026)
Probabilities of AEs in placebo group (%)					
Neutropenia	64.8	51.84	77.76	Beta	Ma et al. (2026)
Leukopenia	50.9	40.72	61.08	Beta	Ma et al. (2026)
PFS	0.85	0.64	1	Beta	Shu et al. (2025)
PD	0.52	0.39	0.65	Beta	Shu et al. (2025)
Disutility due to AEs					
Neutropenia	0.09	0.072	0.108	Beta	Dickerson et al. (2025)
Leukopenia	0.10	0.08	0.120	Beta	Dickerson et al. (2025)
Diarrhea	0.103	0.077	0.129	Beta	Jia et al. (2025)
Anemia	0.073	0.058	0.088	Beta	Jia et al. (2025)
Hypokalemia	0.04	0.032	0.048	Beta	Liu et al. (2023)
Vomiting	0.13	0.104	0.156	Beta	Liu et al. (2023)
Body surface (m^2^)	1.72	1.376	2.064	Normal	Shu et al. (2025)
Body weight (kg)	59	47.2	70.8	Normal	Bao et al. (2022)
Discount rate (%)	4.5	0	5	Fixd	Wu and Liu (2026)

PFS, progression-free survival; PD, progressive disease; AE, adverse event.

### 2.2 Model structure

**FIGURE 1:**
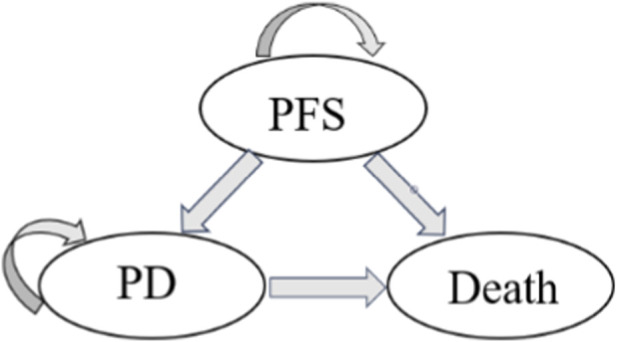
Structure of the established partitioned survival model. PFS, progression-free survival; PD, progressive disease.

In this study, we developed a partitioned survival model using TreeAge Pro 2022 to simulate disease progression in patients with HER2-positive metastatic breast cance (Woods et al., 2020). Patients were stratified into three mutually exclusive health states: PFS, progressive disease (PD), and death. All patients entered the model in the PFS state. As the disease progressed, they could transition from PFS to PD or death, and these transitions were irreversible (Figure 1). The distribution of patients across health states over time was directly estimated based on the areas under the Kaplan-Meier (KM) curves. A cycle length of 3 weeks was adopted in the model, consistent with the treatment regimen of the PHILA study. Given that the median age of patients in the PHILA study was 52 years, the simulation time horizon was set to 48 years, within which most patients were expected to die. The model outputs included total costs, quality-adjusted life years (QALYs), and the incremental cost-effectiveness ratio (ICER). According to the 2025 Edition of China guidelines for pharmacoeconomic evaluation (Wu and Liu, 2026), the willingness-to-pay (WTP) threshold was set at twice the per capita gross domestic product (GDP) of China in 2025 ($27,906/QALY). Both costs and utilities were discounted at an annual rate of 4.5%.

### 2.3 Clinical data

We used GetData Graph Digitizer (version 2.25) to extract data points from the KM curves reported in the PHILA trial. R software (version 4.4.2) was then used to reconstruct individual patient-level data and generate simulated survival curves (Guyot et al., 2012) (Supplementary Figure S1). The reconstructed curves were fitted with a series of parametric distributions, including exponential, Weibull, Gompertz, gamma, generalized gamma, log-normal, and log-logistic distributions. The optimal distribution was selected based on the Akaike Information Criterion (AIC) and the Bayesian Information Criterion (BIC), where smaller values indicated a superior model fit (Supplementary Table S3) (Latimer, 2013). Based on these statistical criteria and visual inspection (Supplementary Figure S2), the log-logistic distribution provided the best fit for the PFS curves in both the pyrotinib and placebo groups. For OS, the log-normal distribution was optimal for the pyrotinib group, whereas the log-logistic distribution was preferred for the placebo group.

### 2.4 Cost and utility

This study was conducted from the perspective of the Chinese healthcare system, considering only direct medical costs. The model included the following costs: drug costs for the study regimen in the PHILA trial and subsequent therapies; follow up management costs, including tumor imaging assessments, laboratory tests, and cardiovascular monitoring; BSC costs; end of life care costs; and expenses related to the management of AEs. Drug cost data were obtained from the median provincial drug procurement prices on the China Yaozhi website (https://www.yaozh.com) in 2025. For the calculation of drug doses, patients were assumed to have an average weight of 59 kg (Bao et al., 2022) and a body surface area of 1.72 m^2^ (Shu et al., 2025). Costs for follow up examinations, as well as management costs for hypokalemia and diarrhea, were derived from local hospital medical charges. Expenses for AE management and BSC were obtained from published literature (Chen et al., 2021; Zhu Y. et al., 2022; Jin and Li, 2025; Zhu et al., 2026). AEs with an incidence of 5% or higher and a severity grade of 3 or higher in the PHILA study were incorporated. These mainly consisted of neutropenia, leukopenia, anemia, hypokalemia, diarrhea, and vomiting (Ma et al., 2026). Consistent with common practice in cost-effectiveness analysis, the cost of AE management was assumed to be incurred only in the first treatment cycle following the event and was incorporated as a one-time cost (Huo et al., 2026). All costs were inflation adjusted to 2025 US dollars using annual inflation rates and converted from Chinese yuan at the 2025 average exchange rate of 7.1429 RMB per USD.

Because health utility values were not reported in the PHILA study, the health utility values for PFS and PD in this study were obtained from previously published literature (Shu et al., 2025). Given that severe AEs may reduce short-term quality of life, we also incorporated disutilities associated with treatment related severe AEs. These parameters were derived from published studies (Liu et al., 2023; Dickerson et al., 2025; Jia et al., 2025). AEs were assumed to occur in the first cycle, and their associated disutilities were deducted from the PFS baseline utility (Shu et al., 2026). Detailed cost and utility parameters are summarized in Table 1.

### 2.5 Scenario analysis

To address uncertainty arising from model assumptions, we performed a series of scenario analyses. First, we conducted a price threshold analysis for pyrotinib to assess how potential price reductions would affect the cost-effectiveness results. Second, because the choice of time horizon can influence long-term costs and outcomes, we varied the simulation horizon to 6, 10, 20, and 30 years and compared the ICERs across these scenarios. Third, we performed survival extrapolations using alternative parametric distributions (Supplementary Table S4) and recalculated the ICERs. Fourth, we assumed that all patients received BSC after disease progression to explore how different second-line treatment strategies would affect the ICER values.

### 2.6 Sensitivity analysis

The robustness of the model was evaluated using one-way sensitivity analysis and probabilistic sensitivity analysis (PSA). In the one-way sensitivity analysis, each parameter was adjusted over a range of ±20% from its baseline value, and the discount rate was varied from 0% to 5%. The results were presented using a tornado diagram. PSA was based on 1000 second-order Monte Carlo simulations, in which all uncertain parameters were randomly sampled to test the stability of the results. According to ISPOR guidelines (Brazier et al., 2019), cost parameters were fitted to a gamma distribution, while utility and probability parameters followed a beta distribution. Patient weight and body surface area were assigned a normal distribution, with standard deviations set at 10% of baseline values. The results were illustrated using incremental cost-effectiveness scatter plots and cost-effectiveness acceptability curves.

## 3 Result

### 3.1 Base-case results

Base-case results are presented in Table 2. Total costs and effectiveness in the pyrotinib arm were $136,400.69 and 5.20 QALYs, respectively, compared with $83,887.55 and 3.89 QALYs in the placebo arm. The ICER was $40,245.81/QALY, which exceeded the WTP threshold of $27,906.03/QALY. Therefore, pyrotinib in combination with trastuzumab and docetaxel was not cost-effective for HER2 positive metastatic breast cancer.

**TABLE 2:** Baseline results.

Strategy	Total cost ($)	Incremental Cost ($)	QALYs	Incremental QALYs	ICER($/QALY)
Pyrotinib Group	136,400.69	52,513.14	5.20	1.3	40,245.81
Placebo Group	83,887.55	—	3.89	—	—

QALY, quality-adjusted life-year; ICER, incremental cost-effectiveness ratio.

### 3.2 Scenario analysis

**FIGURE 2:**
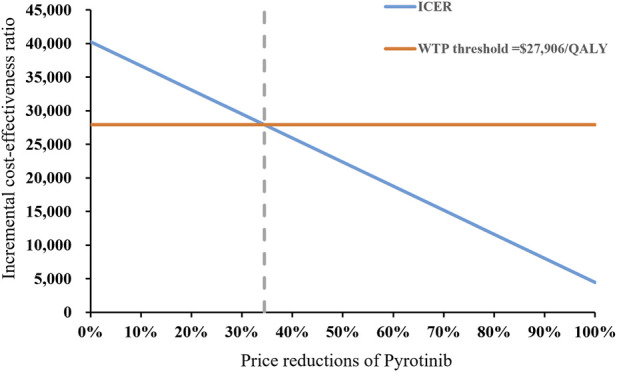
Price reduction threshold analysis of pyrotinib.

The results of the price threshold analysis for pyrotinib are shown in Figure 2. The price needed to be reduced by more than 34.45% to bring the ICER below the WTP threshold of $27,906/QALY, at which point the regimen became cost-effective. Larger price reductions corresponded to lower ICER values. As the simulation horizon increased from 6 to 30 years, both incremental costs and health benefits rose. Pyrotinib had a higher ICER in the short term, but its incremental QALYs increased rapidly after 10 years, leading to a decline in ICER and improved long-term cost-effectiveness. Fitting PFS and OS with suboptimal distributions yielded an ICER of $37,903.40/QALY. Assuming all patients received BSC after progression produced an ICER of $41,991.32/QALY, which exceeded the WTP threshold. Thus, pyrotinib remained not cost-effective in that scenario. Detailed scenario results are presented in Table 3.

**TABLE 3:** Results of scenario analysis.

Scenarios	Strategy	Total cost ($)	Incremental Cost ($)	QALYs	Incremental QALYs	ICER ($/QALY)
Time horizon						
6 years	Pyrotinib	79,579.72	31,962.62	2.88	0.49	65,362.48
	Placebo	47,617.09	—	2.39	—	—
10 years	Pyrotinib	100,744.09	38,975.33	3.74	0.76	51,546.95
	Placebo	61,768.76	—	2.98	—	—
20 years	Pyrotinib	123,946.20	47,612.66	4.69	1.10	43,091.67
	Placebo	76,333.55	—	3.58	—	—
30 years	Pyrotinib	131,971.58	50,810.31	5.02	1.24	41,114.21
	Placebo	81,161.27	—	3.78	—	—
Employing suboptimal distribution						
​	Pyrotinib	130,348.30	59,169.99	4.92	1.56	37,903.40
	Placebo	59,169.99	—	3.36	—	—
Change subsequent treatment						
​	Pyrotinib	145,013.16	54,790.69	5.20	1.30	41,991.32
	Placebo	90,222.46	—	3.89	—	—

QALY, quality-adjusted life-year; ICER, incremental cost-effectiveness ratio.

### 3.3 Sensitivity analysis

**FIGURE 3:**
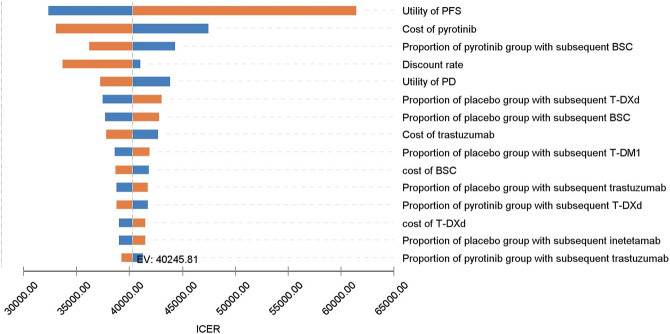
One-way sensitivity analysis tornado diagram of pyrotinib versus placebo. ICER, incremental cost-effectiveness ratio; PFS, progression-free survival; PD, progressive disease; BSC, best supportive care; T-Dxd, trastuzumab deruxtecan; T-DM1, trastuzumab emtansine.

The tornado diagram (Figure 3) showed that the ICER was most sensitive to the utility value of PFS and the price of pyrotinib. Other parameters exerted relatively minor effects, including the proportion of patients receiving BSC, the cost of BSC, the utility value of PD, and the proportion of patients in the pyrotinib and placebo groups receiving second-line therapy with T-DXd, T-DM1, trastuzumab, or pertuzumab. Nevertheless, variations of these parameters within ±20% did not alter the base-case conclusion, nor did they bring the ICER below the predefined WTP threshold.

**FIGURE 4:**
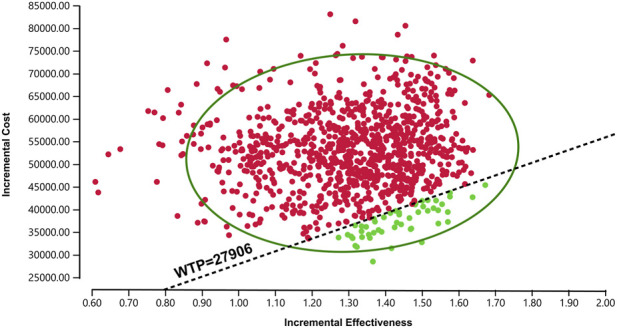
Scatter plot of probabilistic sensitivity analysis of pyrotinib versus placebo. WTP, willingness-to-pay.

**FIGURE 5:**
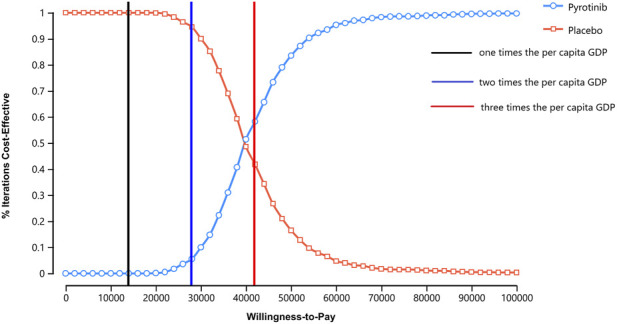
Cost-effectiveness acceptability curve of pyrotinib compared to placebo. GDP, gross domestic product.

PSA showed that all simulated points were located in the northeast quadrant, indicating that pyrotinib was associated with higher costs and greater effectiveness (Figure 4). At a WTP threshold of $27,906/QALY, 93.9% of the simulated points lay above the threshold line, suggesting that pyrotinib was not cost-effective at the prespecified threshold. The cost-effectiveness acceptability curve (Figure 5) revealed that pyrotinib had a 0% probability of being cost-effective at the WTP threshold equivalent to the per capita GDP ($13953/QALY). However, the probability increased to 6.1% when the threshold was raised to twice the per capita GDP ($27,906/QALY). This upward trend continued as the WTP threshold increased further, reaching 57.6% at the WTP threshold set at three times the per capita GDP ($41,859/QALY).

## 4 Discussion

Our base-case analysis showed that compared with the placebo regimen, the pyrotinib combination provided greater health benefits but also increased total treatment costs. The ICER was $40,245.81/QALY, which exceeded the WTP threshold of $27,906/QALY used in this study. Therefore, at the current price level and WTP threshold, the pyrotinib combination was not cost-effective.

Sensitivity analysis showed that the utility value of PFS, the unit price of pyrotinib, the proportion of patients receiving BSC, and the utility value of PD had a relatively large impact on the results. However, variations of all parameters within the predefined ranges did not alter the base-case conclusion, indicating good model stability. The utility values of PFS and PD reflect differences in quality of life across disease stages. Changes in these parameters directly affect QALY gains and ICER estimates, highlighting the importance of health utility parameters in economic evaluation. Given the limited utility data currently available for metastatic breast cancer in China, further measurement and validation of utility values in Chinese populations are needed.

Drug price was also an important factor affecting the results. The price threshold analysis showed that the price of pyrotinib would need to be reduced by more than 34.45% for the ICER to fall below the threshold of twice the per capita GDP. A greater price reduction was associated with a lower ICER. In China’s reimbursement negotiation and formulary inclusion practice, innovative anticancer drugs usually require substantial price reductions to improve accessibility. Previous reports showed that the negotiated price reductions for such drugs ranged from 25.54% to 70.70% (Li C. et al., 2024). Another study reported that the average price reduction of innovative drugs in the eighth round of NRDL negotiations was at least 44% (Sun et al., 2022). Therefore, the 34.45% price reduction scenario has certain practical relevance. Even under the BSC scenario, the pyrotinib combination remained not cost-effective at the current price, suggesting that differences in subsequent treatment costs were not the determining factor for the conclusion of this study. Sensitivity analysis also confirmed that utility values and drug price were the most influential factors, indicating that the economic value of the regimen mainly depended on its health benefits and price level rather than on subsequent treatment costs. Under different simulation horizons, the ICER conclusion remained unchanged, indicating that the model results were not sensitive to the time horizon. When alternative parametric distributions were used, the main conclusion also remained unchanged, further supporting the robustness of the results.

The WTP threshold is an important basis for judging whether a treatment is cost-effective (Zhan et al., 2023). The World Health Organization previously recommended one to three times the per capita GDP as a reference threshold. However, recent studies have suggested that three times the per capita GDP may be too high as a WTP threshold (Woods et al., 2016; Iino et al., 2022; Pichon-Riviere et al., 2023). According to the China Guidelines for Pharmacoeconomic Evaluations (2025) (Wu and Liu, 2026), implemented in December 2025, the WTP threshold was updated to two times the per capita GDP in China. Therefore, this study followed the latest guideline and set the WTP threshold at $27,906/QALY. The guideline also states that, for drugs with prominent innovative value or significant equity improvement, a threshold higher than two times the per capita GDP may be adopted according to decision-making needs. When the WTP threshold was increased to three times the 2025 per capita GDP in China ($41,859/QALY), the pyrotinib combination became cost-effective. This finding indicates that the economic conclusion was influenced to some extent by the level of WTP. Actual decision-making should still consider both affordability and clinical value.

The results of this study were generally consistent with previous studies. Economic evaluations of targeted therapies and novel antibody-based drugs for breast cancer have shown that, although these drugs can improve clinical outcomes, high prices remain the main factor limiting their economic accessibility. Previous studies have shown that pertuzumab, T-DXd, and sacituzumab govitecan are often unlikely to achieve acceptable cost-effectiveness at current prices, and substantial price reductions are usually required before their economic value can improve (Durkee et al., 2016; Dai et al., 2022; Shi et al., 2023; Zhan et al., 2023). The required magnitude of price reduction differs across studies. This may be related to differences in treatment efficacy, regimen composition, adverse event management costs, and WTP thresholds. Overall, these studies suggest that improving the accessibility of novel anti-HER2 and antibody-based drugs in China still requires pricing strategies that match the local ability to pay.

This study also has practical relevance. To our knowledge, this is the first cost-effectiveness analysis of pyrotinib plus trastuzumab and docetaxel for HER2-positive metastatic breast cancer from the Chinese healthcare perspective. In the context of China’s dynamic National Reimbursement Drug List adjustments and reimbursement negotiations, pharmacoeconomic evidence serves as an important basis for drug access and price management. Our findings indicate that under the current price and WTP threshold, the pyrotinib combination is not cost-effective. However, a price reduction through future negotiations could enhance its economic value.

This study has several limitations. First, fitting and extrapolating KM curves using survival models may introduce potential error. External validation of long-term survival extrapolation was not possible because no external long-term follow-up data were available. Therefore, the long-term cost-effectiveness estimates remain uncertain. As longer follow-up data become available, the conclusions of this study should be further validated and updated. Second, the PHILA trial did not report quality-of-life data, and published values from previous studies were used in this analysis, which may affect the local applicability of the results. Third, grade 3 or higher AE parameters were included only in the first cycle, which may have led to underestimation of costs and overestimation of QALYs, although sensitivity analysis showed that these parameters had little impact on the model outcomes.

## 5 Conclusion

Under the drug prices and WTP threshold used in this study, pyrotinib plus trastuzumab and docetaxel is not cost-effective for the treatment of HER2-positive metastatic breast cancer. A reduction in the price of pyrotinib would enhance its cost-effectiveness.

## Data Availability

The original contributions presented in the study are included in the article/Supplementary Material, further inquiries can be directed to the corresponding author.
