# Supplementary material for: Cost-effectiveness analysis of pyrotinib combined with trastuzumab and docetaxel as first-line treatment for HER2-positive metastatic breast cancer in China
Source: Front Pharmacol. 2026 Jun 25;17:1845724. doi: 10.3389/fphar.2026.1845724 (PMC13345860; doi:10.3389/fphar.2026.1845724)
Supplement: Supplementary file 1 [file DataSheet1.docx]

**Supplementary Material**

**1. Supplementary Table S1** The proportion of patients receiving second-line treatment.

**2. Supplementary Table S2** Dosage and administration of subsequent therapeutic agents

**3. Supplementary Tab S3** Comparison of survival models distribution

**4. Supplementary Table S4** The survival model parameters for suboptimal distributions of survival curves

**5. Supplementary Figure S1** Reconstruction of Kaplan Meier survival curve

**6. Supplementary Figure S2** Models simulation visual overall survival curve

**Supplementary Table S1** The proportion of patients receiving second-line treatment.

| **Parameters** | **Estimate** | **Range** | | **Distribution** |
| --- | --- | --- | --- | --- |
|  |  | **Minimum** | **Maximum** |  |
| **Systemic anti-tumor therapy in pyrotinib group** | | | | |
| Trastuzumab | 26.3% | 21.04% | 31.56% | Beta |
| Pertuzumab | 15.5% | 12.4% | 18.60% | Beta |
| Inetetamab | 12.1% | 9.76% | 14.64% | Beta |
| Pyrotinib | 18.5% | 14.8% | 22.20% | Beta |
| Trastuzumab emtansine | 10.1% | 8.08% | 12.12% | Beta |
| Trastuzumab deruxtecan | 6.7% | 5.36% | 8.04% | Beta |
| Chemotherapy | 38% | 30.4% | 45.60% | Beta |
| **Systemic anti-tumor therapy in placebo group** | | | | |
| Trastuzumab | 25.6% | 20.48% | 30.72% | Beta |
| Pertuzumab | 14% | 11.20% | 16.80% | Beta |
| Inetetamab | 18.4% | 14.72% | 22.08% | Beta |
| Pyrotinib | 51.9% | 41.52% | 62.28% | Beta |
| Trastuzumab emtansine | 13.7% | 10.96% | 16.44% | Beta |
| Trastuzumab deruxtecan | 7.8% | 6.24% | 9.36% | Beta |
| Chemotherapy | 62.5% | 50.00% | 75.00% | Beta |

**Supplementary Table S2** Dosage and administration of subsequent therapeutic agents

| **Primary drugs for second-line treatment** | **Usage and dosage** |
| --- | --- |
| Trastuzumab | Initial dose:8 mg/kg; maintenance dose: 6 mg/kg, once every 3 weeks. |
| Pertuzumab | Initial dose: 840 mg; maintenance dose: 420 mg, once every 3 weeks. |
| Inetetamab | Initial dose: 8mg/kg; maintenance dose: 6mg/kg, once every 3 weeks. |
| Pyrotinib | 400 mg per day, once daily. |
| Trastuzumab emtansine | 3.6 mg/kg, once every 3 weeks. |
| Trastuzumab deruxtecan | 5.4 mg/kg, once every 3 weeks. |
| Chemotherapy |  |
| Cyclophosphamide | 600mg/m^2^，once every 3 weeks |
| Doxorubicin | 1.2-2.4mg/kg，once every 3 weeks |
| Paclitaxel Injection | 175mg/m^2^，once every 3 weeks |

**Supplementary Tab S3** Comparison of survival models distribution

|  | **Model** | **AIC** | | **BIC** | |
| --- | --- | --- | --- | --- | --- |
|  |  | **Pyrotinib** | **placebo** | **Pyrotinib** | **placebo** |
| **OS** | Exponential | 1048.800 | 1249.216 | 1052.493 | 1252.896 |
|  | Weibull（AFT） | 1038.655 | 1244.020 | 1046.042 | 1251.380 |
|  | Gamma | 1037.411 | 1243.318 | 1044.799 | 1250.678 |
|  | Generalized gamma | 1036.037 | 1244.965 | 1047.118 | 1256.006 |
|  | Gompertz | 1045.123 | 1248.884 | 1052.511 | 1256.244 |
|  | Log-normal | 1034.244 | 1248.211 | 1041.632 | 1255.572 |
|  | Log-logistic | 1036.833 | 1241.359 | 1044.221 | 1248.719 |
| **PFS** | Exponential | 1734.596 | 1914.462 | 1738.290 | 1918.142 |
|  | Weibull（AFT） | 1736.571 | 1902.499 | 1743.958 | 1909.860 |
|  | Gamma | 1735.813 | 1891.480 | 1743.200 | 1898.841 |
|  | Generalized gamma | 1720.739 | 1870.244 | 1731.820 | 1881.284 |
|  | Gompertz | 1730.930 | 1916.461 | 1738.318 | 1923.822 |
|  | Log-normal | 1718.967 | 1868.747 | 1726.355 | 1876.108 |
|  | Log-logistic | 1717.030 | 1859.186 | 1724.418 | 1866.546 |

AIC: Akaike information criterion; BIC: Bayesian Information Criterion; OS: Overall survival; PFS: Progression-free survival.

**Supplementary Table S4** The survival model parameters for suboptimal distributions of survival curves

| **Group** | **Model** | **Parameters** |
| --- | --- | --- |
| OS of pyrotinib: | Log-logistic | Shape=1.600, scale=87.772 |
| OS of placebo: | Gamma | Shape=1.397, rate=0.0154 |
| PFS of pyrotinib: | Log-normal | meanlog=3.2784, sdlog=1.2869 |
| PFS of placebo: | Log-normal | meanlog=2.441, sdlog=1.2869 |

OS: Overall survival; PFS: Progression-free survival.


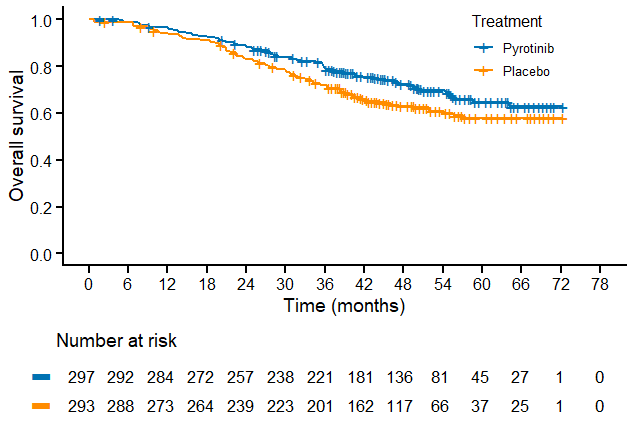


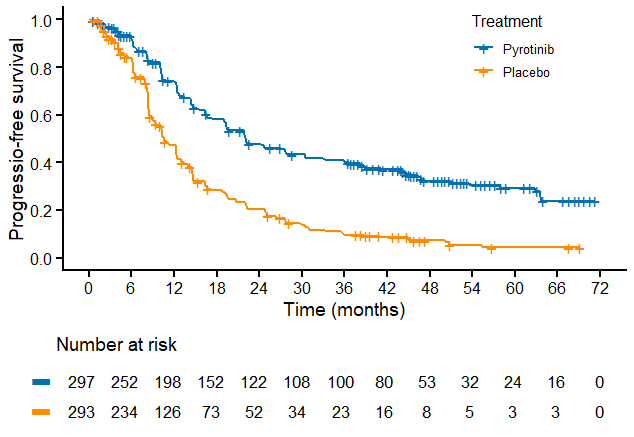


**B**

**A**

**Supplementary Figure S1:** Reconstruction of Kaplan Meier survival curve


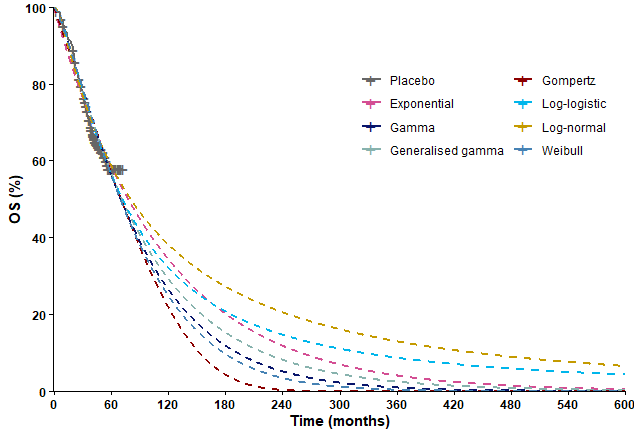


**B**


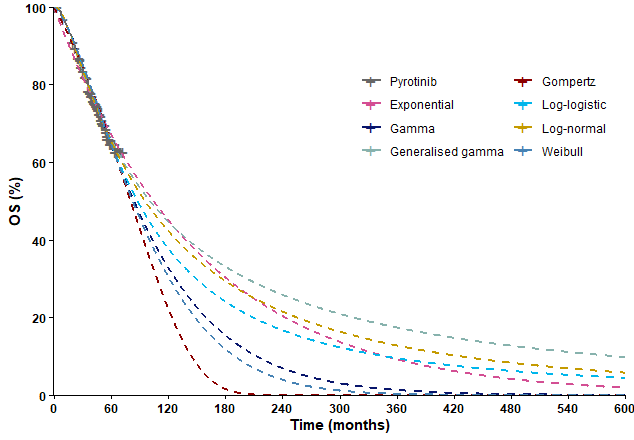


**A**


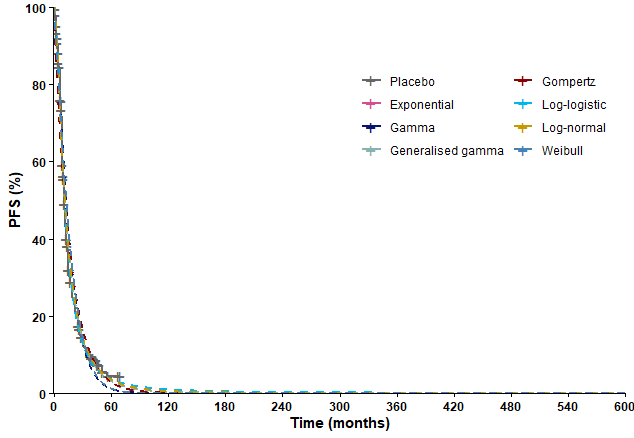


**D**


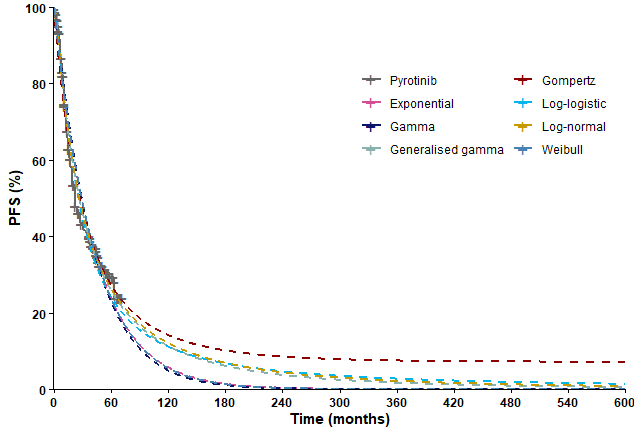


**C**

**Supplementary Figure S2** A Models simulation visual overall survival curve of pyrotinib group; B Models simulation visual overall survival curve of placebo group; C Models simulation visual progression-free survival curve of pyrotinib group; D Models simulation visual progression-free survival curve of placebo group
